# Genome sequences of nicotine-degrading *Pseudomonas* and *Stutzerimonas* strains isolated from nicotine-enriched activated sludge

**DOI:** 10.1128/mra.01355-25

**Published:** 2026-02-27

**Authors:** N. Myers, T. Navaratna, B. Kim, K. S. Myers, D. R. Noguera, T. J. Donohue, J. C. A. Bardwell

**Affiliations:** 1 Department of Molecular, Cellular, and Developmental Biology, University of Michigan 118715, Ann Arbor, Michigan, USA; 2 Howard Hughes Medical Institute, University of Michigan 1259, Ann Arbor, Michigan, USA; 3 Great Lakes Bioenergy Research Center, University of Wisconsin-Madison 282101, Madison, Wisconsin, USA; 4 Wisconsin Energy Institute, University of Wisconsin-Madison 732059, Madison, Wisconsin, USA; 5 Department of Civil and Environmental Engineering, University of Wisconsin-Madison 539348, Madison, Wisconsin, USA; 6 Department of Bacteriology, University of Wisconsin-Madison 205263, Madison, Wisconsin, USA; University of Manitoba, Winnipeg, Canada

**Keywords:** activated sludge, microbiology, nicotine, metabolism, whole-genome sequencing, bacterial genomes

## Abstract

We have isolated seven novel nicotine-degrading *Pseudomonas* and *Stutzerimonas* strains from activated sludge enrichment cultures. These strains were obtained through successive passages of activated sludge microbial communities in M9 minimal media containing nicotine as the sole carbon source, followed by single colony isolation.

## ANNOUNCEMENT

Nicotine metabolic pathways have been extensively studied in bacterial genera, such as *Pseudomonas* and *Arthrobacter* (1). To identify additional nicotine-metabolizing organisms from wastewater treatment environments, we performed enrichment of activated sludge communities using liquid M9 minimal media containing trace metals and 1 g/L nicotine as the sole carbon source. The activated sludge inoculum was collected from the Nine Springs Wastewater Treatment Plant (Madison, WI) and inoculated at 5% into M9 + nicotine media. Cultures were maintained at RT with constant shaking at 200 rpm and passaged once growth stopped increasing (approx. 3–4 days). After six successive passages, individual samples were plated on M9 + nicotine agar plates for single colony isolation. Following single colony isolation, individual isolates were subjected to whole-genome sequencing using Oxford Nanopore Technology (ONT) through Plasmidsaurus (CA, USA).

Single colonies were inoculated into 3 mL of LB and incubated overnight at 37°C with shaking at 200 rpm before pelleting and washing with 1 mL of DPBS, and then resuspending in 500 μL of Zymo DNA/RNA Shield. DNA was extracted using the Zymo Quick-DNA Miniprep Plus Kit before being sent for sequencing. Libraries were constructed with v14 library prep chemistry, and sequencing was performed on R10.4.1 flow cells without primers.

Genome assembly was conducted by Plasmidsaurus using the following pipeline: the bottom 5% of reads were removed using Filtlong v0.2.1 (2) to eliminate low-quality sequences. Total reads ranged from 56,000 to 214,000 reads. An initial assembly was created with Miniasm v0.3 (3), followed by assembly with Flye v2.9.1 (4) using parameters optimized for high-quality ONT reads, and coverage was downsampled to 100× for samples with raw coverage over 100×. The Flye assembly was polished with Medaka v1.8.0 (5) to improve base-level accuracy. Genome completeness and contamination were assessed using CheckM v1.2.2 (6). Taxonomic classification was performed using GTDB-Tk v2.6.1 (7). All software was run with standard parameters unless otherwise noted.

**Fig 1:**
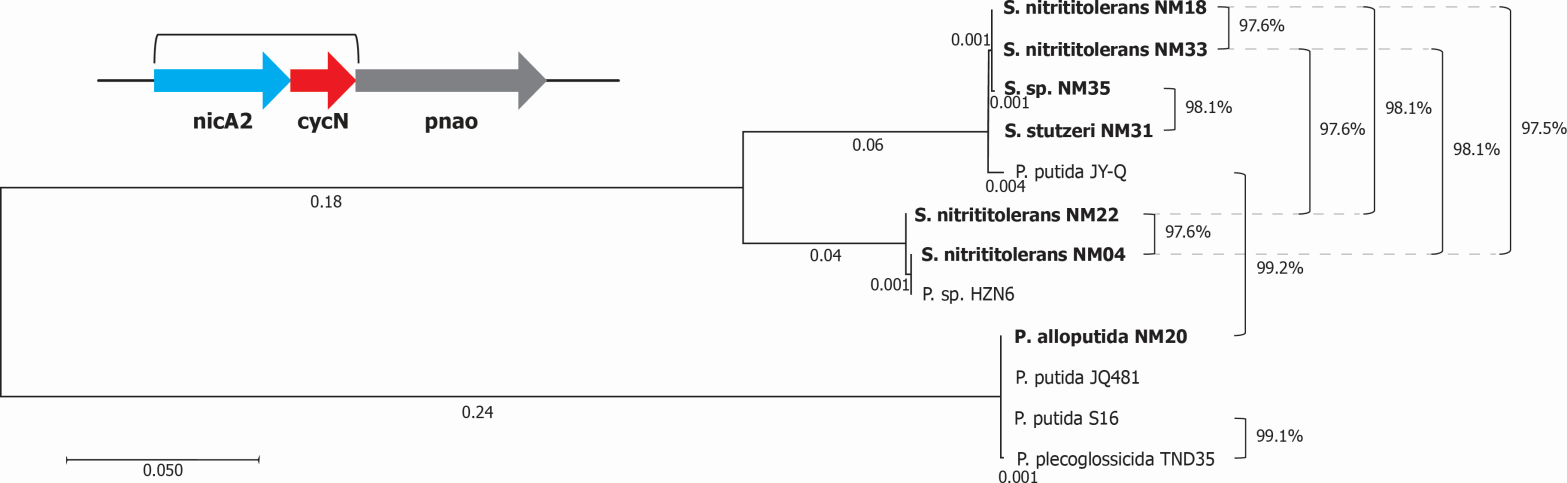
Phylogenetic analysis of nicotine-degrading *Stutzerimonas* and *Pseudomonas* strains. Maximum likelihood (ML) phylogenetic tree showing the relationship of the newly sequenced strains (in bold) to previously characterized nicotine-degrading *Pseudomonas* strains. The tree was constructed using the portion of the nicotine degradation operon containing *nicA2* through *cycN* or their homologous sequences (1,892 bp). In addition to the HZN6 nicotine degradation cluster (JN391188.2), reference strains were used: S16 (GenBank accession NC_015733), JY-Q (NZ_CP011525), JQ581 (NZ_CP050951), and TND35 (JOJY00000000). The tree was built using MEGA12 (14) by first performing a multiple sequence alignment with Muscle (15) run on default parameters. An ML tree was then made with a Kimura 2-parameter, gamma-distributed tree with 95% site coverage partial deletion and nearest-neighbor interchange. Numbers represent branch lengths with the scale bar showing five substitutions per 100 sites. Percentages indicate % average nucleotide identity (ANI) across the entire genome calculated using dRep with a primary clustering threshold of 0.95 and a secondary clustering threshold of 0.99 (16). All pairwise ANIs were calculated, and percentages >95% are shown. ANI was not calculated for HZN6 because it is only the nicotine degradation cluster and not the whole genome.

The genome sequences of seven nicotine-degrading isolates were obtained, with genome sizes of approximately 4 to 4.5 Mb for all isolates, except for NM20 (approximately 6 Mb). GC content ranged from 62 to 63%. Read N50 values ranged from 5 to 10 kb. Reads were then assembled into draft assemblies each consisting of one linear contig. Assembly coverage ranged from 60× to 100×. Genome completeness ranged from 99.9 to 100%, while contamination levels were 1% or less for all strains. GTDB-Tk (7) analysis classified NM20 as *Pseudomonas alloputida* and the other isolates as members of the genus *Stutzerimonas* (Table 1). The genomes contain putative nicotine degradation operons similar to those in other *Pseudomonas* species (1). Phylogenetic analysis with MEGA and dRep (Fig. 1) positioned these isolates alongside previously characterized nicotine-degrading *Pseudomonas* strains (8–12). The new isolates tended to have similar nicotine degradation operons yet differed significantly at the whole genome level. Additionally, mobile elements flanking the nicotine degradation operons were identified, suggesting horizontal gene transfer events similar to that of the *Arthrobacter* nicotine cluster (13).

**TABLE 1:** Genome characteristics of novel nicotine-degrading strains

Strain	Completeness (%)	Contamination (%)	Length (bp)	Read N50	GC content (%)	Raw coverage (×)	Assembled coverage (×)	Annotated genes	Total bp sequenced (bp)	Total reads	Taxonomy	Accession no.
NM04	100	0.14	4,373,013	5,183	63	117	99.5	4,089	512,208,282	178,604	*Stutzerimonas nitrititolerans*	SAMN53033551
NM18	100	0.68	4,312,926	9,199	63	64	60.6	4,070	275,919,868	56,500	*Stutzerimonas nitrititolerans*	SAMN53033552
NM20	99.9	1.02	6,039,682	10,329	62	103	98.1	5,565	626,050,030	109,035	*Pseudomonas alloputida*	SAMN53033553
NM22	100	0.31	4,284,175	6,737	63	145	99.8	3,983	623,899,169	159,921	*Stutzerimonas nitrititolerans*	SAMN53033554
NM31	100	0.55	4,499,974	6,405	62	106	99.9	4,273	478,998,232	127,627	*Stutzerimonas stutzeri*	SAMN53033555
NM33	100	0.27	4,262,259	7,740	63	224	100	3,980	956,031,597	214,229	*Stutzerimonas nitrititolerans*	SAMN53033556
NM35	100	0.55	4,510,989	7,522	62	61	58.7	4,213	279,443,766	65,020	*Stutzerimonas* sp.	SAMN53033557

## Data Availability

The genome sequences have been deposited in figshare at https://doi.org/10.6084/m9.figshare.30676094.v2. Raw sequencing reads have been deposited in GenBank under BioProject accession number PRJNA1355196.
